# Nb/Sn Liquid-Solid Reactive Diffusion Couples and Their Application to Determination of Phase Equilibria and Interdiffusion Coefficients of Nb-Sn Binary System

**DOI:** 10.3390/ma15010113

**Published:** 2021-12-24

**Authors:** Jiali Zhang, Jing Zhong, Qin Li, Lijun Zhang

**Affiliations:** State Key Lab of Powder Metallurgy, Central South University, Changsha 410083, China; jializhang@csu.edu.cn (J.Z.); zhongjingjogy@gmail.com (J.Z.)

**Keywords:** liquid-solid diffusion couple, Nb-Sn system, Nb_3_Sn, phase equilibria, diffusion, HitDIC

## Abstract

Nb_3_Sn plays an irreplaceable role in superconducting parts due to its stable performance under high field conditions. Accurate phase equilibria and interdiffusion coefficients are of great significance for designing novel Nb_3_Sn superconductors. However, the related experimental information is still in a state of scarcity because of the difficulty in fabrication of Nb-Sn alloys caused by the large difference in melting points of Nb and Sn. In this paper, a simple but pragmatic approach was first proposed to prepare the Nb/Sn liquid-solid reactive diffusion couples (LSDCs) at 1100 °C and 1200 °C, of which the phase identification of the formed layer and the measurement of composition-distance profiles were conducted. The formed layer in Nb/Sn LSDCs was confirmed to be Nb_3_Sn compound. While the measured composition profiles were employed to determine the phase equilibria according to the local equilibrium hypothesis and the interdiffusion coefficients with an aid of the latest version of HitDIC software. The determined phase equilibria of Nb_3_Sn, (Nb) and liquid show good agreement with the assessed phase diagram. While the calculated interdiffusion coefficients and activation energy for diffusion in Nb_3_Sn are consistent with both experimental and theoretical data in the literature. Moreover, the growth of the formed Nb_3_Sn layer in Nb/Sn LSDCs was also found to be diffusion controlled. All the obtained phase equilibria and interdiffusion coefficients are of great value for further thermodynamic and kinetic modeling of the Nb-Sn system. Furthermore, it is anticipated that the presently proposed approach of fabricating liquid-solid reactive diffusion couple should serve as a general one for various alloy systems with large differences in melting points.

## 1. Introduction

Due to its high superconducting critical temperature Tc = 18 K, high superconducting critical magnetic field Hc = 27T (4 K) and stable performance [1], Nb_3_Sn superconductor has been of great importance in high field working environments since the 1960s [2]. Tokamak device, used in nuclear-fusion research for magnetic confinement of plasma, is the core unit of both the International Thermonuclear Experimental Reactor (ITER) project and the China Fusion Engineering Test Reactor (CFETR) project. Nb_3_Sn acts as the vital toroidal field coil in Tokamak devices [3]. However, as one of A15 type intermetallic compounds in the cubic lattice system [4], Nb_3_Sn owns the poor plasticity and toughness, and can be easily damaged, resulting in destruction of superconductivity. In order to improve the properties/performance of Nb_3_Sn-based parts and even explore novel Nb_3_Sn-based alloys, accurate phase equilibria and diffusion coefficients [5,6] are the prerequisites for understanding the Nb-Sn and its related systems. Unfortunately, a very large difference in melting points between Nb (2468 °C) and Sn (231.89 °C) makes it extremely difficult to fabricate the Nb-Sn alloys. To overcome this problem, researchers have made many attempts, and developed several techniques for preparing Nb-Sn alloys, including the powder-in-tube process [7], the bronze process [8], the internal tin process [9], the restacked rod process [10], the powder metallurgy method [11], and so on. Even so, the experimental data on phase equilibria and diffusion coefficients of the Nb-Sn binary system are still in a state of scarcity.

Up to now, only about 10 groups of researchers have performed experimental measurement of phase equilibria of the binary Nb-Sn system [12,13,14,15,16,17,18,19,20]. Based on the limited experimental phase equilibrium information, Toffolon [18] performed a CALPHAD (CALculation of PHAse Diagram) [21] thermodynamic assessment of Nb-Sn system in 1998. During the assessment, Toffolon [18] adopted the conclusion of Massalski [17] and Shunk [22] that Nb_3_Sn would not be stable below 796 °C. However, on account of the enthalpy of formation of Nb_3_Sn determined by drop calorimetry and other new literature data [13,14,15], it was believed that Nb_3_Sn can exist stably down to the room temperature. Then in 2002, Toffolon [19] updated the thermodynamic assessment of binary Nb-Sn system accordingly. Although the calculated phase equilibria of binary Nb-Sn system according to the thermodynamic descriptions by Toffolon [19] are in general agreement with the limited experimental data [12,13,14,15,16,20], it is still necessary to provide more accurate experimental phase equilibrium data for further validation. While for the diffusion coefficients in Nb-Sn binary, there is only one report from Wallach [23], who obtained the interdiffusion coefficients of Nb_3_Sn from the ternary Cu-Nb-Sn system measured by two different methods, i.e., the Nb-bronze multi-layered thin-film composites and the multi-filamentary composites. Moreover, the data from Wallach [23] only cover the low-temperature range between 350 °C and 800 °C. Again, accurate experimental diffusion coefficients of the binary Nb-Sn system, especially at high temperatures, are in urgent need.

Consequently, the major objectives of this paper are: (i) to fabricate the Nb/Sn liquid-solid reactive diffusion couples (LSDCs) at 1100 °C and 1200 °C. Here, a simple but pragmatic approach is to be proposed for preparing the Nb/Sn LSDCs, of which the melting points of two end-members show a large difference; and (ii) to apply the prepared Nb/Sn LSDCs to determine the accurate phase equilibria and interdiffusion coefficients of the binary Nb-Sn system at the corresponding temperatures. The phase equilibria are to be measured based on the classic local equilibrium hypothesis, while the interdiffusion coefficients of the compound Nb_3_Sn will be evaluated by using the HitDIC (High-throughput Determination of Interdiffusion Coefficients) software [24,25,26,27], based on the measured composition-distance profiles over the interdiffusion zones in Nb/Sn LSDCs.

## 2. Materials and Methods

**Figure 1:**
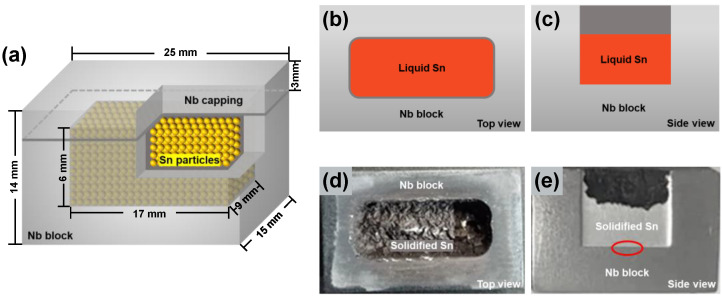
Geometry of Nb/Sn LSDC: (**a**) Schematic diagram for the assembled Nb/Sn LSDC; (**b**) top view and (**c**) side view of Nb/Sn LSDC during the heat treatment. Typical photos of physical sample for the Nb/Sn LSDC after annealing at 1200 °C for 2 h: (**d**) top view and (**e**) side view.

The raw materials for preparing Nb/Sn LSDCs were Nb blocks (25 × 15 × 14 mm) with a purity of 99.99% (wt.%) and Sn particles (1~2 mm in diameter) with a purity of 99.99% (wt.%). The Nb block was fixed on the leveling workbench for blind hole processing. An Nb cap (25 × 15 × 3 mm) was first cut from the Nb block by means of the wire-electrode technique, and then a blind hole (17 × 9 × 6 mm) was dug by the drill bit in the center of the remaining Nb block. Considering that the high-speed rotation of the drill bit may generate a large amount of heat, causing the slight oxidation on the surface of the Nb block, it is thus necessary to polish the inner surface of the blind hole after machining to remove the oxide layer and level the inner surface. After that, Sn particles were filled into the hole, and the Nb block was welded and sealed with the Nb cap. The assembled Nb/Sn LSDC is schematically shown in Figure 1a.

Two identical Nb/Sn diffusion couples were put into the corundum boats equipped with quartz cotton. The corundum boats containing the samples were preheated for a few minutes at the furnace port and then subjected to annealing at 1100 °C for 2.33 h and 1200 °C for 2 h in a high-temperature vacuum tube furnace (GSL1700X, Hefei Kejing Materials Technology Co., Ltd., Hefei, China), respectively. There are three reasons for choosing the annealing temperatures as 1100 °C and 1200 °C. First, according to the assessed phase diagram [19], only three stable phases (bcc, liquid and Nb_3_Sn) exist in the Nb-Sn binary system between 911 °C~2152 °C. Such simple phase equilibria are beneficial for the study on thermodynamic and diffusion properties related to the Nb_3_Sn phase. Second, no experimental studies on the phase equilibria in Nb-Sn binary system around 1100 °C. Third, the reported interdiffusion coefficients of Nb_3_Sn phase only cover the low-temperature range (350 °C~800 °C), and more information on interdiffusion coefficients of Nb_3_Sn phase at higher temperatures is highly needed. Moreover, the formation of Nb_3_Sn phase was clearly observed in Nb-bronze multi-filamentary composites after annealing at 700 °C for only 1 h in the work of Ref. [28]. Therefore, the annealing time at 1100 °C and 1200 °C was chosen to be around 2 h for the formation of Nb_3_Sn phase with sufficient thickness in Nb/Sn LSDCs. At both 1100 °C and 1200 °C, the Nb block keeps the solid state, while the Sn particles are melted into the liquid state. The schematic diagrams for top view and side view of Nb/Sn LSDC are displayed in Figure 1b,c. After annealing, the Nb/Sn LSDCs were quickly quenched in liquid nitrogen. Subsequently, each Nb/Sn LSDC was cut into five pieces, and metallographically polished for the subsequent analysis of interdiffusion zones over the Nb/Sn interface. The photos of the physical samples of the Nb/Sn LSDCs after quenching are presented in Figure 1d,e. The optical microscopy (OM), scanning electron microscope (SEM), electron probe micro-analysis (EPMA), and electron backscattered diffraction (EBSD) techniques were used to analyze the microstructure and composition of the Nb/Sn LSDCs.

## 3. Results and Discussion

### 3.1. Phase Equilibria

**Figure 2:**
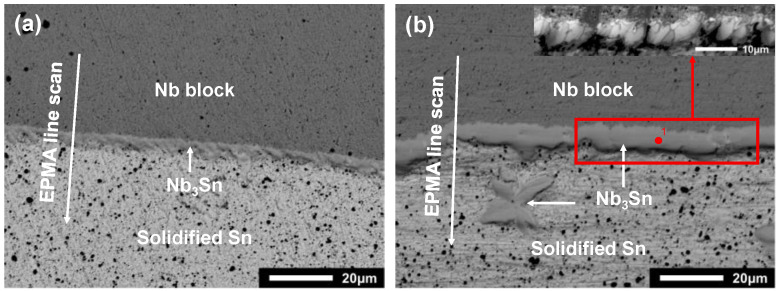
BSE images of Nb/Sn LSDCs annealed (**a**) at 1100 °C for 2.33 h, and (**b**) at 1200 °C for 2 h.

**Figure 3:**
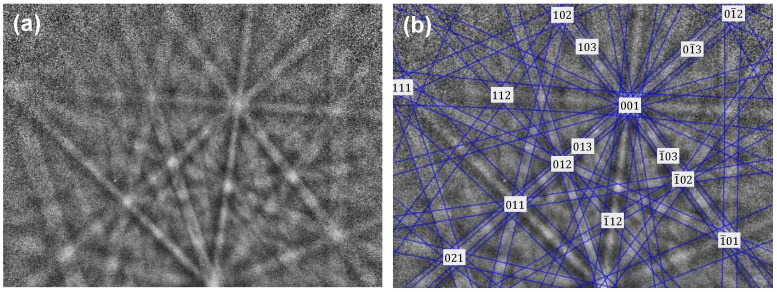
(**a**) EBSD pattern of Nb_3_Sn phase and (**b**) its index calibration in Nb/Sn LSDC annealed at 1200 °C for 2 h. The pattern was acquired with the voltage of 20 kV, and the specimen tilting angle was set to 70°.

According to the Nb-Sn binary phase diagram assessed by Toffolon [19], three phases are stable over the temperature range of 911~2152 °C, i.e., bcc, Nb_3_Sn and liquid. The microstructure of the Nb/Sn LSDCs annealed at 1100 °C and 1200 °C taken from the position of the red circle in Figure 1e are respectively shown in Figure 2a,b. As can be observed in the figures, one apparent layer formed over the interdiffusion zone across the Nb/Sn interface at both 1100 and 1200 °C. The composition of the formed layer was measured by means of the EPMA technique, while its crystal structure (at point 1 in Figure 2b) was characterized by using the EBSD technique, as shown in Figure 3. Both EPMA and EBSD results indicated that the layer formed over interdiffusion zone is the Nb_3_Sn phase, which corresponds well with the Nb-Sn phase diagram assessed by Toffolon [19]. Moreover, the average size of Nb_3_Sn grains annealed at 1200 °C for 2 h was measured to be 4.36 ± 0.85 μm.

**Figure 4:**
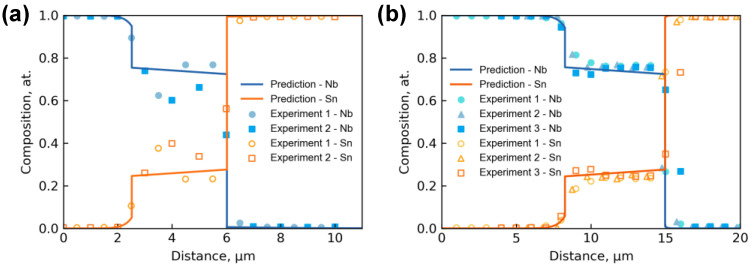
Experimental composition-distance profiles in the Nb/Sn LSDCs annealed (**a**) at 1100 °C for 2.33 h, (**b**) at 1200 °C for 2 h, compared with the corresponding model-predicted composition-distance profiles by HitDIC. Note that the caption “Experiment 1, 2 and 3” in the figures denotes the three separate measurements of composition profiles for each annealed Nb/Sn sample.

As also indicated in Figure 2, the EPMA scans of the compositions in Nb/Sn LSDCs across the entire diffusion zone perpendicularly to the diffusion interface were performed, and the obtained composition-distance profiles are displayed as scattered points in Figure 4a,b. Here, it should be noted that two EPMA runs were conducted for Nb/Sn LSDC at each temperature. Based on the measured composition-distance profiles, the average thickness of the Nb_3_Sn phase layer can be evaluated to be 6.63 ± 1.86 μm at 1200 °C for 2 h and 3.43 ± 0.62 μm at 1100 °C for 2.33 h.

**Figure 5:**
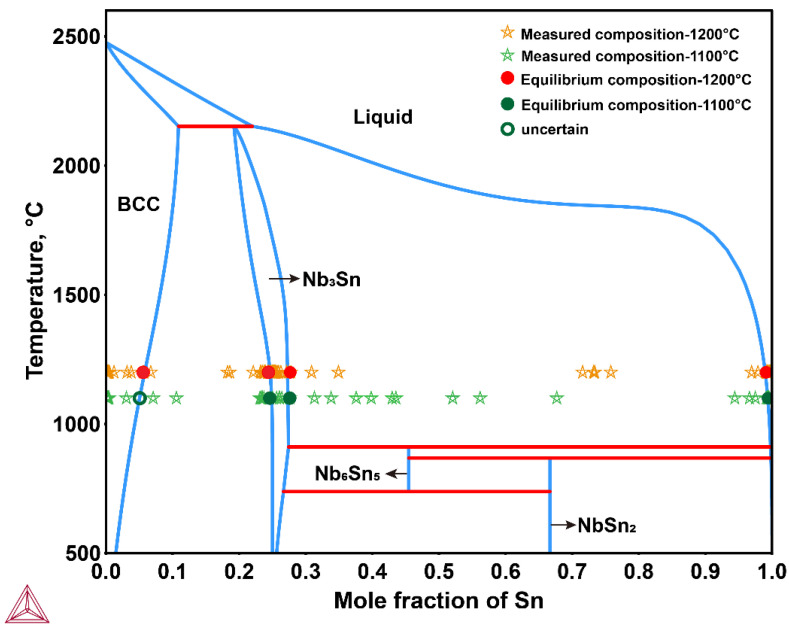
Comparison between the experimental composition profiles measured in this study and the Nb-Sn binary phase diagram calculated according to the thermodynamic parameters assessed by Toffolon [19].

The measured concentration profiles of Nb/Sn LSDCs at two temperatures were then superimposed on the Nb-Sn binary phase diagram from Toffolon [19], as presented in Figure 5. It is worth mentioning that there should be no two-phase mixture regions in binary diffusion couples because they are thermodynamically forbidden. Thus, based on the local equilibrium hypothesis at the phase interfaces, the equilibrium compositions of phases could be obtained by extrapolating the measured concentration profiles to the phase interfaces. The concentration profiles of Nb_3_Sn and liquid phases can be clearly seen in Figure 5, and their equilibrium compositions at 1100 and 1200 °C were determined and marked by solid circles in Figure 5 and summarized in Table 1. As can be clearly seen in the figure, the determined homogeneity range of Nb_3_Sn compound at both 1100 °C and 1200 °C are in good agreement with the assessed phase equilibria by [19]. Moreover, it is astonishing to see a general agreement between the determined solubility limits of Nb in liquid (Sn) at both 1100 °C and 1200 °C and the assessed ones [19] exist, though there should be some further phase transformations from the remaining liquid to solids during quenching of LSDCs. For the solubility of Sn in (Nb) phase, the determined value at 1200 °C is in good consistency with the assessed one [19], as displayed in Figure 5. However, the solubility of Sn in (Nb) phase at 1100 °C cannot be accurately determined due to the relatively scattered composition profile of Nb/Sn LSDC at 1100 °C. Instead, a rough value between 3.1 at.% and 7.1 at.% was proposed for the solubility of Sn in (Nb) phase at 1100 °C, which is still close to the assessed one by [19]. Based on Figure 5 and the above analysis, the phase equilibria in binary Nb-Sn system at both 1100 °C and 1200 °C determined by the present LSDCs and predicted by thermodynamic assessment [19] should be reliable.

### 3.2. Interdiffusion Coefficients

Based on the experimental composition-distance profiles yielded in the interdiffusion zones of Nb/Sn LSDCs, the interdiffusion coefficients were extracted using the latest version of HitDIC software [24,25,26] originally developed under the framework of pragmatic numerical inverse approach [29,30]. By simplifying the diffusion processes in reactive diffusion couple to be a one-dimensional moving boundary problem, the evolution of composition c_n_ for the *n*-th phase and interface position s_n_ between the n-th phase and the (*n* + 1)-th phase described as

(1) $$\frac{\partial c_{n}}{\partial t}=\frac{\partial }{\partial r}\left(D_{n}\frac{\partial c_{n}}{\partial r}\right)$$

(2) $${D_{n-1}\frac{\partial c_{n-1}}{\partial r}|}_{r=s_{n}^{-}\left(t\right)}-{D_{n}\frac{\partial c_{n}}{\partial r}|}_{r=s_{n}^{+}\left(t\right)}=\left(c_{n+1}-c_{n}\right)\frac{ds_{n}}{dt}\;\;\mathrm{at}\;r=s_{n}\left(t\right)$$

where $D_{n}$ are the interdiffusion coefficients to be recovered by means of the numerical inverse method. That is, with the setting of a diffusion couple, i.e., initial compositions of terminal alloys, diffusion time, and component-distance profile, an inverse routine based on optimization is used to iteratively evaluate a suggested set of interdiffusion coefficients for all the solution phases and intermetallic compounds. At each iteration, deviation between the prediction and experimental observations can be evaluated, while suggestions for an alternative set of interdiffusion coefficients may be provided according to the employed optimization methods, i.e., the genetic algorithm. The optimization processes can be terminated once the satisfactory prediction result towards the experimental observation is obtained, and the related set of interdiffusion coefficients can therefore be taken as the determined results. Among the calculation processes, the local equilibrium assumption is applied for adjacent phases in the reactive diffusion couple.

The measured composition-distance profiles were used to evaluate the interdiffusion coefficients for the Nb-Sn system at 1100 °C and 1200 °C, respectively. The predicted composition-distance profiles using HitDIC are also displayed as the solid lines in Figure 5. As can be seen in the plots, good consistency between the predicted composition-distance profiles and the experimental data exists for the two investigated temperatures. Here, only the interdiffusion coefficients of Nb_3_Sn compound are to be evaluated. That is because (i) during the quenching, the liquid Sn near the interdiffusion zone and diffused Nb may form the NbSn_2_ phase, resulting in the Sn content in the diffusion zone near the liquid side lower than the theoretical value, and (ii) the spatial resolution of EPMA makes the measured composition points close to (Nb) side insufficient for evaluating the interdiffusion coefficient of bcc phase especially at 1100 °C.

**Figure 6:**
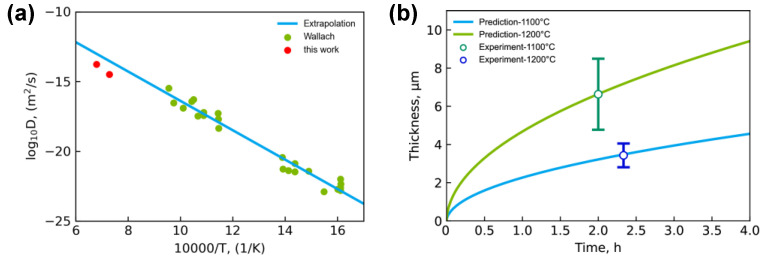
(**a**) Comparison between the presently evaluated interdiffusion coefficients of Nb_3_Sn at 1100 °C and 1200 °C and the low-temperature data from Wallach [23]; (**b**) Model-predicted thickness of the Nb_3_Sn layer as function of annealing time for the Nb/Sn LSDCs, compared with the experimental data in the present work.

Figure 6a shows the presently extracted interdiffusion coefficients of Nb_3_Sn from Nb/Sn LSDCs at 1100 °C and 1200 °C (i.e., 4.66 × 10^−15^ m^2^∙s^−1^ and 1.85 × 10^−14^ m^2^∙s^−1^), which generally follow the same trend as the low-temperature data from multi-layered thin-film composites and multi-filamentary composites [23]. Based on presently extracted interdiffusion coefficients at 1100 °C and 1200 °C, the pre-exponential factor and the activation energy for diffusion in Nb_3_Sn compound can be obtained by fitting to the Arrhenius equation:

(3) $$D=D_{0}\exp\left(-\frac{Q}{RT}\right).$$

The obtained pre-exponential factor of the Nb_3_Sn phase *D*_0_ in Equation (4) is 1.80 × 10^−6^ m^2^∙s^−1^, while the activation energy *Q* is 232.04 kJ∙mol^−1^. The presently evaluated activation energy for Nb_3_Sn compound shows very good agreement with the data from the kinetics measurements (i.e., 248.71 kJ∙mol^−1^) by [31] and ab initio density functional theory calculations (i.e., 269.79 kJ∙mol^−1^) by [32], validating the reliability of the presently obtained interdiffusion coefficients of Nb_3_Sn compound.

Moreover, with interdiffusion coefficients calculated by HitDIC and the simultaneous diffusional growth of Nb_3_Sn layer [33], the average thickness of the Nb_3_Sn phase layer was determined as a function of the annealing time, which can be expressed as the following equation,

(4) $$l=k{\left(\frac{t}{t_{0}}\right)}^{n},$$

where *l* is the average thickness of the Nb_3_Sn phase layer, *t* is the annealing time, and *t_0_* is the unit time. As shown in Figure 6b, we obtained *k* = 2.28 μm and *n* = 0.51 for the Nb/Sn LSDC annealed at 1100 °C, and *k* = 4.69 μm and *n* = 0.51 for the Nb/Sn LSDC annealed at 1200 °C, with the experimental thickness data locating precisely on the predicted profile. Such a fact indicates that the growth of the Nb_3_Sn phase layer is controlled by diffusion.

**Figure 7:**
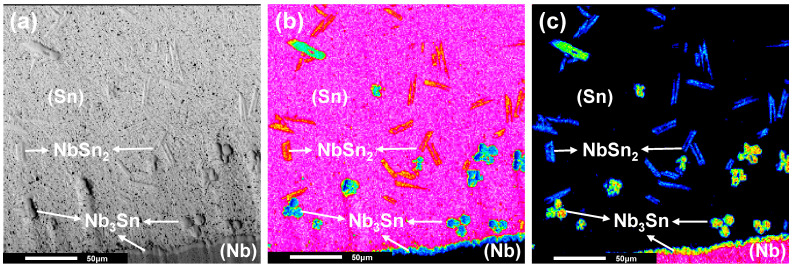
(**a**) BSE image, (**b**) mapping distribution of Sn, and (**c**) mapping distribution of Nb of Nb/Sn LSDC annealed at 1200 °C for 2 h.

It should be noted in Figure 6a that the interdiffusion coefficients of Nb_3_Sn obtained in this work are a bit lower than the values extrapolated from low temperature data [23]. To explore the probable reasons for this case, the interface of the Nb/Sn liquid-solid reactive diffusion couple was further characterized by experiments. The element mapping characteristics over the diffusion zone were analyzed using the EPMA map analysis, as shown in Figure 7. The result shows that the Nb_3_Sn phase layer formed across the Nb/Sn interface, while some free Nb_3_Sn particles existed in the (Sn) region. The reason is considered that the Nb element diffused into melted Sn during the annealing process, and some Nb_3_Sn particles formed and remained in the solidified Sn region after the quench. There are also some lath-like microstructures distributing in the solidified (Sn) region, and that might be due to the composition segregation in solidified liquid that resulted in the formation of the NbSn_2_ phase. Because of the peritectic reactions, L + Nb_3_Sn→NbSn_2_ and L + NbSn_2_→(Sn), a fraction of generated Nb_3_Sn phase reacted with the remained liquid, which can make the measured thickness of the Nb_3_Sn phase layer smaller than the actual one, thus lowering the interdiffusion coefficients of the Nb_3_Sn phase.

## 4. Conclusions

A simple but pragmatic approach was proposed to fabricate the Nb/Sn LSDCs, which were subjected to annealing at 1100 °C and 1200 °C for 2.33 h and 2 h, respectively. It was found that apparent interdiffusion zones formed across the Nb/Sn interface in both LSDCs. The interdiffusion zone was then comprehensively characterized by EPMA and EBSD techniques. Both composition and crystal structure results confirm that the formed layer at the Nb/Sn interface is the Nb_3_Sn phase.

The equilibrium homogeneity range of Nb_3_Sn, solubility limits of (Nb) and liquid phases at 1100 °C and 1200 °C were determined, and the results are in general agreement with the Nb-Sn binary phase diagram assessed by [19].

The interdiffusion coefficients of the Nb_3_Sn phase at 1100 °C and 1200 °C were evaluated by HitDIC software to be 3.22 × 10^−15^ m^2^∙s^−1^ and 1.73 × 10^−14^ m^2^∙s^−1^, respectively. The evaluated diffusion properties of compound Nb_3_Sn, including the interdiffusion coefficients and activation energy, are consistent with the corresponding experimental and theoretical values in the literature. Moreover, the relation between the model-predicted thickness of the Nb_3_Sn layer and the annealing time of Nb/Sn LSDCs was also observed to be in good agreement with the experimental data, indicating the growth of Nb_3_Sn layer in Nb/Sn LSDCs is diffusion controlled.

The presently proposed approach for preparing Nb/Sn LSDCs is anticipated to be a universal one for various alloy systems with large differences in melting points. Furthermore, combined with the local equilibrium hypothesis and HitDIC software, the corresponding phase equilibria and diffusion coefficients can be accurately determined simultaneously.

## Figures and Tables

**Table 1:** Equilibrium compositions for different phases measured from the Nb/Sn LSDCs.

Temperature (°C)	Equilibrium Phases		Equilibrium Compositions (at.% Sn)	
	Phase 1	Phase 2	Phase 1	Phase 2
1100	bcc	Nb_3_Sn	3.1~7.1	24.6
	Nb_3_Sn	liquid	27.6	99.4
1200	bcc	Nb_3_Sn	5.7	24.4
	Nb_3_Sn	liquid	27.6	99.1

## Data Availability

Data sharing not applicable.
